# A case series on TNF-α inhibitors for APS- induced high-risk pregnancies

**DOI:** 10.1017/cts.2025.10097

**Published:** 2025-08-29

**Authors:** Ran An, Xiaolei Wang, Ligui Xiong, Yanqi Yang, Peiling Li

**Affiliations:** 1 Department of Obstetrics and Gynecology, The Fourth Affiliated Hospital of Harbin Medical University, Harbin, Heilongjiang, P.R. China; 2 Department of Immunology and Rheumatology, Cangzhou Hospital of Integrated TCM-WM Hebei, Cangzhou, Hebei, P.R. China; 3 Immune Reproductive Clinic, Hubei Provincial Hospital of TCM Affiliated to Hubei University of Traditional Chinese Medicine, Wuhan, Hubei, P.R. China; 4 Department of Obstetrics and Gynecology, the Second Affiliated Hospital of Harbin Medical University, Harbin, Heilongjiang, P.R. China

**Keywords:** Antiphospholipid syndrome, tnf-α, inhibitors, dangerous pregnancy, gestational weeks

## Abstract

We deliberated a case report of seven cases to investigate whether inhibitors of tumor necrosis factor-α (TNF-) could reduce pregnancy dangers caused by antiphospholipid syndrome (APS). Patient 1 was diagnosed with NC-OAPS and Hashimoto, Patient 3 was with SN-APS and Hashimoto, Patient 2, 3, 4 were with SN-APS, Patient 5 and 6 were with OAPS, and Patient 7 was with OAPS and PCOS. Patient 4 took the longest period to report the disappearance of symptoms (7 days), followed by patients 1 and 5, and lastly, 2, 3, 6 and 7; after treatment, TNF-α decreased to varying degrees in 7 patients, among which Patient 1, 3, 6, 7 reached the ideal level (< 8.1) and Patient 5 reached the highest level (123.04); Patient 6 and 7 were ongoing pregnancies. The fetuses were born to the desired gestational age except the fetus from Patient 1. A total of 5 patients underwent cesarean delivery. The average height of the newborns was 48.20 cm and the average weight was 2.50 kg. The Apgar scores ranged between 8 and 10. The ongoing pregnancies as a limitation of the dataset. Collectively, we found that TNF-α Inhibitors could prolong gestational period.

## Introduction

APS refers to an autoimmune condition known to cause pregnancy complications to line autoimmune thrombocytopenia, thrombosis, preeclampsia, fetal loss, or fetal growth restriction [1]. Obstetric complications caused by APS include oligohydramnios, recurrent miscarriage, premature and early delivery, fetal distress, neonatal or fetal thrombosis, intrauterine growth restriction, eclampsia, preeclampsia, HELLP syndrome, placental insufficiency, and venous or arterial thrombosis are the severe outcomes of APS in pregnancy [2]. The occurrence of these complications threatens both maternal and fetal health. The severity of these complications calls for an effective intervention to save pregnancies: the life of pregnant women and their infants.

Despite a lack of strong evidence on the treatment of APS in pregnancy, TNF-α inhibitors have been reported as possible and potent interventions [3,4]. Different TNF-α inhibitors have been reported to be well-tolerated. Notably, neither malformations in newborns nor implantation failures or miscarriages in the first trimester have been reported [3]. TNF-α inhibitors have been associated with few adverse effects among treated pregnant women, like miscarriage, which poses a significant health concern among women [5]. Miscarriage, alongside other pregnancy complications, has prompted the formulation of multiple interventions to save the lives of pregnant women and neonates. According to the revised Sapporo criteria for classification of the antiphospholipid syndrome, the disease is characterized by thrombosis, pregnancy complications, or both in patients with persistent antiphospholipid antibodies (lupus anticoagulant, anticardiolipin antibodies, or anti-β2GPI antibodies [2]. The persistence of Antiphospholipid antibodies is known to cause venous and arterial thrombosis and loss of pregnancy. Anticardiolipin antibodies IgG or IgM (ELISA), anti-*β*-2glycoprotein-I antibodies IgG or IgM (ELISA), and lupus anticoagulants (functional essays) are the main Antiphospholipid antibodies [6–9]. The presence of lupus anticoagulants is the strongest risk factor for both arterial and venous thrombosis in APS. In several studies, it has been demonstrated that the risk of arterial and venous thrombosis increases with the number of positive tests for aPL, with the highest risks in patients with both LAC, aCL and anti-*β*2GPI antibodies, so-called “triple positive patients.” According to the current guideline of APS, heparin and low-dose aspirin are the standard of care for preventing miscarriage[10]. However, there is the 20–30% of failed pregnancies after the treatment. Therefore, it is needed to search for entirely new therapeutic strategies for poor APS.

There is an increase in the number of childbearing females treated with TNF-*α* inhibitors. In many patients, TNF-*α* inhibitors have been reported to be fast-acting, well-tolerated, and highly active. After decades of safety concerns issues, the Food and Drug Administration carried out a study and classified TNF-α inhibitors “Category B” in pregnancy. This classification suggested that animal studies in whom the studies had been performed reported no adverse effects or safety concerns [11]. In corroboration with FDA’s declaration and reports from other studies, some investigations support the potency of TNF-α inhibitors and provide a rationale for their indication. An investigation by Clowse et al. reported TNF-*α* inhibitors neither caused teratogenicity effects nor increased fetal death, in contrast with the general population [12], as they have been linked with decreased miscarriages. A recent study reported 18 patients with poor APS that received TNF-α inhibitor treatment 70% of patients developed the good obstetric results after anti-TNF-α treatment[3]. In this case serious, we analyze and report the effects of TNF-α inhibitors in pregnancy. We will focus on TNF-α inhibitors’ potency in saving pregnancy complications caused by APS. We will focus on TNF-α inhibitors’ ability to relieve contractions. Hence, reducing miscarriages. The scope of the present study is investigating general outcomes of TNF-α inhibitors in the dangers of pregnancy caused by APS.

## Case descriptions

The basic features of the study participants in the present investigation include age, diagnostic time, height, weight, Body Mass Index (BMI), pre-treatment assessment outcomes, diagnosis, and the respective overall BMI status. First, the study participants were examined in different health facilities and at different times. We evaluated 7 cases and reported their features as age, year of diagnosis, weight, height and the BM (Table 1). The average age and BMI were 32 years and 26.72 kg/m^2^. The reference point of BMI was 18.5 kg/m^2^ to 23.9kg/cm^2^ (Chinese Population Obesity Criteria). 7 women in two institutions were treated with TNF-*α* inhibitors.

**Table 1.** Basic features and clinical data of the study specimen

Patient	Patient 1	Patient 2	Patient 3	Patient 4	Patient 5	Patient 6	Patient 7
Year of diagnosis	2021	2020	2019	2018	2018	2018	2019
age	27	30	33	38	34	30	33
Height (cm)	162	160	168	165	153	167	161
Weight (kg)	53	60	60	50	80	106	81
BMI (kg/m^2^)	20.2	23.4	31.3	18.4	34.2	38.4	31.2
Conception	NC	NC	NC	ART-FET	NC	NC	NC
TNF-α inhibitors therapy	Certolizumab pegol*1 dose	Certolizumab pegol*1 dose	Adalimumab*1 dose	Aspirin*2 dose	Adalimumab*1 dose	Certolizumab pegol*1 dose	Certolizumab pegol*1 dose
Mode of delivery	Cesarean delivery	Cesarean delivery	Cesarean delivery	Cesarean delivery	Cesarean delivery	–	–
Treatment during pregnancy	LWMH	LWMH and aspirin (low-dose)	LWMH and aspirin (low-dose)	LWMH	LWMH and aspirin (low-dose)	LWMH	LWMH
TNF-α inhibitor usage time (weeks)	8 + 2	17 + 2	27	26	16	13 + 2	15 + 3
Gestational age at birth(weeks)	33 + 1	38	37 + 1	37	32 + 5	24 + 1	16 + 4
Fetal weight (grams)	2300	2350	3400	2250/2250	1950	Ongoing pregnancy	Ongoing pregnancy
The fetus number	single birth	single birth	single birth	gameplay pregnancy	single birth	Monocyesis	Monocyesis
Previous obstetric history	MC*6	MC*5	MC*5	FL*3	FL*1	MC*4	FL*2

**Note:** FL= Fetal loss ( > 10weeks); MC= Miscarriage ( <10weeks): NC= Natural conception; ART= Artificial reproductive technology; FET= Frozen embryo transfer.

## Diagnostic assessment

The diagnostic criteria for APS are based on the 2006 Sapporo International Classification Standard Sydney Revision. The diagnosis of APS has to meet at least 1 clinical criterion and at least 1 laboratory criterion, besides, high antibody titer measurements must be carried out with an interval of at least 12 weeks. The clinical criteria include (1) vascular thrombosis: ≥1 arterial, venous or small vessel thrombosis of any tissue or organ (clearly found on imaging or histopathology), and there is no vasculitis in the vascular wall of the thrombus site; (2) Pathological pregnancy: ① ≥1 unexplained fetal arrest after 10 weeks of pregnancy (confirmed by ultrasound or direct examination). (2) ≥1 preterm birth caused by eclampsia, severe preeclampsia or severe placental dysfunction before 34 weeks of pregnancy. (3) ≥3 consecutive abortions of unknown cause within 10 weeks of pregnancy (excluding women’s hormone levels, anatomical abnormalities and chromosomal abnormalities in both men and women).Laboratory criteria include (1) ≥2 positive lupus anticoagulant; (2) ≥2 medium/high titer IgG and/or IgM type aCL in serum or plasma (value >40GPL/MPL or titer>99th percentile of the general population); (3) ≥2 times of medium/high titer IgG and/or IgM β2GPI antibody in serum or plasma (titer >99th percentile of the general population). Pathological pregnancies such as recurrent miscarriage, eclampsia, and placental insufficiency are called obstetric APS (OAPS). Some OAPS patients only meet typical clinical criteria, atypical laboratory criteria or typical laboratory criteria, atypical clinical criteria, which is called atypical OAPS (non-criteria OAPS, NC-OAPS). Patients in this research are classified according to OAPS, NC-OAPS and SN-APS.

## Exclusion

In this work, patients with previous arterial or venous thrombosis were excluded. Also, patients whose withabnormal foetal karyotype were excluded. Patients with active hepa titis B virus (HBV) and cytomegalovirus (CMV) infection, chronic hepatitis C virus (HCV), human immunodeficiency virus (HIV1-2) infection, and latent tuberculosis infection were also excluded.

## TNF-*α* inhibitor treatment

Patients were administrated with adalimumab at the dose of 40 mg/ 2 weeks, starting with 2 doses before embryo transfer. All patients were also put on pro gesterone 800 mg/day until week 12 of pregnancy.

## Case reports

Before the administration of TNF-*α* inhibitors, we examined seven women from two institutions: Hubei Provincial Hospital of TCM Affiliated to Hubei University of Traditional Chinese Medicine, Cangzhou Hospital of Integrated TCM-WM Hebei. The other pre-treatment outcomes (Number of lost pregnancies and method of conception, etc.) were compared with post-treatment observations.

Table 1 summarizes the clinical data of the examined women. The clinical data included the intervention during pregnancy, the TNF*α* inhibitors therapy, mode of delivery, TNF-*α* inhibitors usage in weeks and the gestational week, outcome measurements were taken and limited to the particular week indicated the in the table above. Inconsistent pregnancy outcomes could be attributed to the different treatment during pregnancy. Cases 1, 4, 6 and 7 were treated with LWMH, whereas cases 2, 3 and 5 were treated with LWMH and aspirin (low-dose). Generally, the overall intervention in each case was unique since treatment, period of treatment and therapy varied across the board. We observed different fetal weight. In contrast to the normal fetal weight, the ideal point of reference of 2.5 to 4.0 kgs. We did not observe fetal weight in cases 6 and 7 as they were ongoing pregnancies. Nonetheless, case 1, 2, 4, and 5 reported fetal weight below the required threshold: 2.30, 2.35, 2.25/2.25 and 1.95 kgs, respectively, case 3 reached the ideal threshold: 3.4 kgs. Also, TNF-α inhibitor usage time (weeks) period varied in the seven cases. The least period of use of TNF-α inhibitor was 8 + 2, in case 1, and the highest were cases 3 and 4, 27 and 26 weeks.

## Outcomes of the case reports

These 7 cases focused the 4 study outcomes: term births, premature births, abortion, and extant children. We focused on preterm miscarriage. Participants in the two study centers reported successful sustained pregnancy after treatment. Considering that these conditions often prevent carrying the pregnancy to full term, the use of TNF-a inhibitors appears to help prevent preterm birth. The outcomes of these cases can be contrasted with post-treatment outcomes to establish the dangers averted by TNF-*α* inhibitors. Premature miscarriage are severe and unwanted incidences following and associated with pregnancy. Reduced premature pregnancies prolonged gestational weeks after treatment with TNF-α inhibitors. This was key to protecting maternal and neonatal health.

Table 2 reports detailed specimen data: age, pregnancy BMI, pre-existing diseases, endocrinology, Antiphospholipid syndrome, thrombophilia and other rheumatic diseases. These are the key to determining maternal health status that inform whether pregnancy could be compromised or safety. Patient 1 was diagnosed with NC-OAPS, patient 5, 6, 7 were diagnosed with OAPS, and patient 2, 3, 4 were diagnosed with SN-APS (Table 1). Cases 1 and 3 were diagnosed with Hashimoto and case 7 with PCOS.

**Table 2.** Detailed information of study specimen

Patients	Patient 1	Patient 2	Patient 3	Patient 4	Patient 5	Patient 6	Patient 7
Ages	27	30	33	38	34	30	33
Pre-pregnancy BMI	19.5	22.4	20.8	18.1	33.0	38.4	31.2
Preexisting disease	None	None	None	None	None	None	None
Endocrinology	Hashimoto	–	Hashimoto	–	–	–	PCOS
Antiphospholipid syndrome	NC-OAPS	SN-APS	SN-APS	SN-APS	OAPS	OAPS	OAPS
Other rheumatic diseases	no	no	no	no	no	no	no
Thrombophilia	no	no	no	no	no	no	no

Table 3 summarizes the differences between pre- and post-treatment TNF-α’s levels in the blood (The measurable level was <8.1), timing of TNF-α inhibitors intervention and the results. Patient 4 took the longest period, 7 days, to report the disappearance of symptoms, followed by patients 1 and 5, and lastly, 2, 3, 6 and 7. The disappearance of symptoms of a risk pregnancy shows the effectiveness of the interventions. A decision can be made from these observations on the rationale of administering TNF-α inhibitors. Using Wilcoxon matched-pairs signed rank test, TNF-α level at post-treatment was lower than that at pre-treatment (pre-treatment vs post-treatment:140.48(56.03, 218.37) vs 10.71 (6.60, 29.94), *p* = 0.031). After treatment, TNF-α decreased to varying degrees in all 7 patients, among which cases 1, 3, 6, 7 reached the ideal level (< 8.1) and case 5 reached the highest level (123.04). The gestational age at birth was reported, cases 6 and 7 were ongoing pregnancies, case 1 did not reach the ideal gestational age (37–42 weeks), and the rest of the fetuses were born to the desired gestational age.

**Table 3.** TNF-α’s levels in the blood, timing of TNF-α inhibitors intervention and the results

		TNF-α (<8.1);			
Patients	TNF-α inhibitors usage time (weeks)	Pre-treatment	Post-treatment	Time of symptom disappearance (day)	Gestational age at birth (weeks)
1	8 + 2	42.2	7.81	2	33^+1^
2	17 + 2	97.5	13.6	1	38
3	27	230	6.2	1	37^+3^
4	26 + 5	183.46	35.39	7	37
5	16	271.86	123.04	2	32^+5^
6	13 + 2	<2	<2	1	24 + 1 Ongoing pregnancy
7	15 + 3	13.34	4.6	2	16 + 4 Ongoing pregnancy

Table 4 summarizes post-treatment outcomes in the seven cases, where the effect of TNF-*α* inhibitors was measures after delivery. Five out of the seven cases underwent cesarean delivery. The average height of the newborns was 48.20 cm, whereas the average weight was 2.50 kg. The biggest takeaway from this experiment regards the difference in the gestational period within which the newborns were delivered. The seven cases reported different gestational weeks, which had different additional days. Unfortunately, only 2 cases (all of them are ongoing pregnancy) of the 7 cases was not associated with cesarean delivery. The Apgar scores ranged between 8 and10, whereas the height and the weights of the delivered children varied across the cases.

**Table 4.** A summary of the post-treatment pregnancy outcomes in the seven cases

Patient	Gestational week	male	Female	Cesarean delivery	Height (cm)	Body weight(kg)	Apgar score
1	33 + 1	–	1	1	50	2.30	8
2	38	1		1	45	2.35	9
3	37 + 3	–	1	1	51	3.40	10
4	37	–	2	1	48	2.25/2.25	10
5	32 + 5	1		1	47	1.95	10
6	24 + 1, Ongoing pregnancy	–	–	–	NA	NA	NA
7	16 + 4, Ongoing pregnancy	–	–	–	NA	NA	NA

## Discussion

Immune function abnormalities, immune inflammation, immune system activation or dysfunction occupy an important position in the occurrence of abortion and premature birth. Shortened pregnancy and the loss the pregnancy through resulting preterm miscarriage is a significant health concern. TNF-*α* inhibitors have been floated as potential interventions against early contractions that lead to premature miscarriage.

Tumor necrosis factor-alpha (TNF-*α*) is an inflammatory cytokine belonging to Th1 lymphocytes, which regulates the secretion of prostaglandin E2, human chorionic gonadotropin, serum human placental prolactin, progesterone and renin in the placenta, affects placental formation and embryonic development, and is a cytokine with pro-inflammatory and prothrombotic effects. The elevated level of TNF-α is highly related to pregnancy loss and preeclampsia and contributes to abnormal production of cytokines and chemokines in syncytiotrophoblasts[13]. Currently, TNF-α inhibitors have showed the therapeutic role in poor pregnancy. For example, Zhong et al reported the pregnancy outcome of 10 patients with potential pregnancy loss after TNF-α inhibitor treatment. They found the hCG trajectory of patients were resumed a normal level after TNF-α inhibitor treatment, with no adverse fetal[5]. Meyer et al determined the continuation of anti-TNF therapy after 24 weeks of pregnancy is benefited for prematurity, while not affecting neonatal outcomes and serious infections in the offspring[14]. Thus, anti-TNF-α therapy may decrease the poor outcome of pregnancy. In this work, the effects of TNF-α inhibitor was investigated.

Table 1 summarizes the basic features of the study participants involved in the investigation. Further, Table 4 provides detailed maternal data that affects pregnancy safety. BMI of the participants involved in the present study did not affect the outcomes significantly. The differences in gestational weeks were a fundamental observation. The seven cases reported different gestational weeks. Table 4 summarized the gestational weeks and the birth weights of the newborns in each case. Different TNF-α inhibitor therapy were administered for different weeks within the gestation period. This could account for much of the differences in the observations. Nonetheless, the major outcome regards successful gestation since we did not observe preterm miscarriage. With reference to Case 2 and 4, we established that gestational weeks were dependent on the length of use of TNF-α inhibitors: gestational weeks increased according to the period of use of the TNF-*α* inhibitors. The intervention was found to prolong gestation that would end in normal delivery, safely. Table 3 summarizes the effect of TNF-α on the blood levels of the patients, we found that the levels of TNF-α were reduced to varying degrees in all patients, which can also be considered as a positive effect of the intervention. One study was carried out to establish TNF-α inhibitors’ effectiveness against APS for pregnancy-related complications, the study reported that the outcomes had been enforced by many laboratories and medical institutions where women are diagnosed with such complications. Positive maternal and neonatal outcomes were found among women who received TNF-α inhibitors treatment [15]. We focused on gestational weeks, founded that TNF-α inhibitors have a positive effect on prolonging gestational weeks.

The emphasis put on the different gestational weeks and additional days does not imply that they should or ought to be similar. However, they ought to have been relative to each other. Individual differences and characteristics can be put across to cancel out the difference in gestational week’s factor. Despite the major differences in gestational weeks, we found that existing literature contest that TNF-α inhibitor treatment contributes to the attainment of an average birth weight of approximately 2.5 ∼ 4.0 kg [16,17]. We found that all newborns approach the fetal birth weight, 2.5 to 4.0 kg. We did not measure the birth weight in case 6 and 7 as it was ongoing pregnancy. Cases 1, 2, 3, 4, 5 were found with a birth weight (Table 4). These observations support the argument that TNF-α inhibitors could save pregnancy dangers by increase gestational weeks.

We found positive outcomes of TNF-α inhibitors intervention during pregnancy. Of great significance was the increased birth weight, increase gestational weeks, and successful pregnancy. Except cases 6 and 7, none of the 5 cases reported preterm miscarriage. Cases 6 and 7 reported ongoing pregnancy. We would expect 7 successes with the probability of 0.061 if the underlying expected rate of success in these patients is 67% [3]; In fact, underlying success rate was 71% (5 successes in 7 patients) in this work, and we expected 7 successes with the probability of 0.091. Thus, TNF-α inhibitor might decrease the risk of poor outcome of patients with APS-induced high-risk pregnancy. We observed a concomitant decrease in TNF-a serum levels when treating patients with TNF-α inhibitors. This was associated with an increased pregnancy duration, highly suggestive that TNF-α inhibition can extend gestation and reduce the risk of preterm delivery. However, future case-control studies are needed to determine the efficacy of this approach. This may be a fundamental outcome of the intervention since maternal and neonatal life was protected. Often, preterm miscarriages lead to the loss of neonatal life or can require a cesarean section to save maternal or neonatal life [13,18]. In our analysis, we did not observe a case where a cesarean procedure was required to save neonatal or maternal life. Instead, this is all due to maternal subjective factors (e.g., pain, fear), so we can categorize this outcome as a specific factor.

## Limitation

Our case report provides some interesting evidence on the treatment of adverse pregnancy with inhibitors, but the sample size may affect the reliability of the conclusions to some extent. Therefore, we plan to conduct a prospective, multicenter case-control study in the future to validate the findings of this report with a sufficient sample size. Our study reported a total of 7 cases. Among them, 5 cases did not report preterm birth (delivered via cesarean section), while the remaining 2 cases did not report pregnancy outcomes due to still being in an ongoing pregnancy state, which indicated the use of TNF-α inhibitors might not cause preterm delivery in pregnant individuals. Therefore, TNF-α inhibitors show promise for clinical treatment in female patients with APS under pregnancies. However, there was lack of a control group or a comparative group with standard treatment since this work is a case report. The results might overstate the effectiveness of TNF-α inhibitors. In future, we will perform a case-control study with a control group or a matched historical comparison group to strengthen causal inferences.

## Conclusion

This present study reported the possible therapeutic role of TNF-α inhibitors’ effectiveness in reducing pregnancy dangers caused by APS based on key pregnancy outcomes. TNF-α inhibitors might save neonatal and maternal health, which will be further verified in future studies.

## Data Availability

The datasets used or analyzed during the current study are available from the corresponding author on reasonable request.
